# Molecular Radiobiology in Non-Small Cell Lung Cancer: Prognostic and Predictive Response Factors

**DOI:** 10.3390/cancers14092202

**Published:** 2022-04-28

**Authors:** Javier Peinado-Serrano, Amancio Carnero

**Affiliations:** 1 Instituto de Biomedicina de Sevilla, IBIS, Hospital Universitario Virgen del Rocio, Consejo Superior de Investigaciones Científicas, Universidad de Sevilla, Avda. Manuel Siurot s/n, 41013 Seville, Spain; jvrr18@gmail.com; 2 CIBERONC, Instituto de Salud Carlos III, 28029 Madrid, Spain; 3 Department of Radiation Oncology, Hospital Universitario Virgen del Rocio, Avda. Manuel Siurot s/n, 41013 Seville, Spain

**Keywords:** NSCLC, radiotherapy, biomarkers, prognostic and predictive signature

## Abstract

**Simple Summary:**

The identification of prognostic and predictive gene signatures of response to cancer treatment (radiotherapy) could help in making therapeutic decisions in patients affected by NSCLC. There are multiple proposals for gene signatures that attempt to predict survival or predict response to treatment (not radiotherapy), but they mainly focus on early stages or metastasis at diagnosis. In contrast, there have been few studies that raise these predictive and/or prognostic elements in nonmetastatic locally advanced stages, where treatment with ionizing radiation plays an important role. In this work, we review in depth previous works discovering the prognostic and predictive response factors in non-small cell lung cancer, specially focused on non-deeply studied radiation-based therapy.

**Abstract:**

Non-small-cell lung cancer (NSCLC) is the leading cause of cancer-related death worldwide, generating huge economic and social impacts that have not slowed in recent years. Oncological treatment for this neoplasm usually includes surgery, chemotherapy, treatments on molecular targets and ionizing radiation. The prognosis in terms of overall survival (OS) and the different therapeutic responses between patients can be explained, to a large extent, by the existence of widely heterogeneous molecular profiles. The identification of prognostic and predictive gene signatures of response to cancer treatment, could help in making therapeutic decisions in patients affected by NSCLC. Given the published scientific evidence, we believe that the search for prognostic and/or predictive gene signatures of response to radiotherapy treatment can significantly help clinical decision-making. These signatures may condition the fractions, the total dose to be administered and/or the combination of systemic treatments in conjunction with radiation. The ultimate goal is to achieve better clinical results, minimizing the adverse effects associated with current cancer therapies.

## 1. Introduction

### 1.1. Ionizing Radiation as an Oncological Treatment

The clinical benefit of ionizing radiation has its sole counterpart in surgical treatment, and these two therapeutic weapons are responsible for the possible cures in cancer. Radiotherapy is administered in at least 60% of patients affected by a neoplasm, with constantly improving tolerance and safety profiles and excellent clinical results in the radical, complementary, or palliative clinical scenario. Currently, antineoplastic treatment is being considered as personalized medicine. Personalization aims to offer the patient individualized treatment adapted to their physical characteristics and the characteristics of their particular neoplasm. However, the oncological treatments used, both in localized and metastatic stages, are far from optimal in terms of individualization. Although there are several neoplasms for which we can offer more precise therapeutic alternatives, we continue to use general treatments, such as surgery, ionizing radiation, and chemotherapy, as standards. Individualized treatment for systemic therapies will require focusing the therapeutic approach on specific molecular targets. However, customization of radiotherapy treatment focuses mainly on the application of immobilization elements adapted to the anatomy of each patient. This can enable a gain in precision, as well as technological advances in diagnostic imaging, including computed tomography (CT) for treatment planning and its fusion with positron emission tomography (PET) and magnetic resonance imaging (MRI). Radiotherapy treatment in its different modalities was known to be effective long before the molecular basis for its effectiveness was discovered. This molecular effect, focused directly and/or indirectly on the damage to nuclear and/or mitochondrial DNA, is only the tip of the iceberg of the biological effect of this powerful therapeutic weapon. Radiotherapy treatment has successfully withstood the appearance of numerous waves of therapeutic alternatives that threaten to replace it as the second most important therapy after surgery. Instead, it has managed to evolve toward therapeutic personalization, now offering great precision and an unprecedented tolerance profile. Radiotherapy offers some patients the possibility of long-term cure with an excellent toxicity profile. Despite this, we still need a deeper understanding of the molecular bases that govern the different therapeutic effects after ionizing radiation.

### 1.2. Classical Radiobiology: The 5 “Rs” That Explain the Response to Ionizing Radiation

Several phenomena have been described that condition the disparate responses to ionizing radiation. This has laid the groundwork for the use of different treatment schemes depending on the histological subtype and stage of the disease. These factors, commonly known as the 5 “Rs” of radiobiology, are applicable to both healthy and tumor tissues:

Intrinsic radiosensitivity: Two tumors of different histological lineages show a very different therapeutic response. A good example of this is the case of melanoma, considered radioresistant to standard fractionations, and low-grade follicular lymphoma, whose curative treatment requires very low doses of radiation. Likewise, two tumors considered to be of the same histological lineage also sometimes show very different therapeutic responses. Deepening the study of this variable opens the field of molecular alterations typical of each tumor cell. Alterations in the signaling pathways that mediate proliferation, damage repair or programmed cell death, among others, determine the wide range of responses observed in the clinic. The study of in vitro intrinsic radiosensitivity is carried out by means of the clonogenicity assay, obtaining the parameter surviving fraction at 2 Gy (SF2) that allows the comparison of results of different histological subtypes and the prediction of the potential clinical response of the said tumor. The study of the molecular factors involved in this response is one of the pillars of modern molecular radiobiology.

Reoxygenation: The first studies suggesting an important role of tissue oxygenation in the response to radiotherapy treatment date back to 1909, when Schwarz [1] showed that ionizing radiation had a dampened effect on the skin if it was compressed externally, thus reducing the flow of blood in the irradiated area. In 1910, Müller showed greater responses to treatment upon increasing blood flow with diathermy [2]. In 1953, Gray et al. [3], as pioneers in radiobiology, published their studies on the effect of hypoxia on the response to ionizing radiation using an in vitro fibroblast model and an in vivo mouse model. They postulated that the oxygen concentration conditions the chemical response induced in the cells after radiation. In that same year, the double helix structure of DNA was described by Watson and Crick [4]. Based on this, direct and indirect damage (by oxygen-nitrogen free radicals) to DNA was postulated to be one of the main mechanisms of the action of ionizing radiation. This damage is influenced by hypoxia-reoxygenation, which is the cornerstone of physical and molecular radiobiology. In 1955, the presence of hypoxic areas in some squamous cell carcinomas of the lung and their potential relationship with the response to radiation were described [5]. Since then, many neoplastic histological subtypes have been studied, with hypoxia being an independent prognostic factor in cervical cancer [6,7], head and neck carcinomas [8] and soft tissue sarcomas [9] among others. In the last 20 years, knowledge of the molecular mechanisms that mediate the response to oxygen levels and their role in the phenomena of initiation, maintenance, proliferation, metastasis, and response to oncological treatments has increased significantly [10].

Redistribution: The response to ionizing radiation differs depending on the phase of the cell cycle. Pioneering studies at the end of the 1960s placed the late phase of cell cycle synthesis (S phase) as the phase with the least sensitivity to radiation [11]. In contrast, the late phase of G2 and mitosis are the most sensitive to it. Likewise, through the fractionation of the dose, a redistribution toward more sensitive phases of the cell cycle can be achieved. This could explain the greater benefit of such fractional schemes. The factors responsible for cell cycle-dependent response variations are multiple and not yet fully determined, suggesting that the ability to repair damage by homologous recombination (HR), is one of the main reasons behind these differences.

Repair: Since direct and/or indirect damage to DNA is ultimately responsible for the effects of ionizing radiation, repairing this damage is key to understanding the therapeutic response. After exposure to a treatment fraction, there are three subtypes of potentially inducible damage: (1) lethal damage, which is not repairable and generally involves cell death (of both tumor and normal cells); (2) sublethal damage, which involves the generation of molecular alterations that are properly recognized by damage sensors and can be repaired; and (3) potentially lethal damage, which, if not properly repaired, can lead to a lethal event. Neoplastic cells sometimes have mutations, such as the inhibition of TP53 or other sensors, such as ATM and BRCA, which lead to the abnormal or failed recognition of induced DNA damage. This, added to an imperfect repair machinery, ultimately leads to the appearance of successive clones with little or no response to treatment.

Repopulation: When fractionated radiotherapy treatment is advanced on a given tumor, a cellular subpopulation with pluripotential properties can regenerate the damaged tumor itself [12]. These cellular phenomena are described in the majority of tumors and partially justify the early local relapses observed in the clinic. This is a consequence of a greater resistance to treatment of these pluripotent clones (cancer stem cells (CSCs)). It is usually overcome by reaching a sufficient total absorbed radiation dose and not stopping the fractionated treatment before its theoretical completion. Radiation is highly effective in neoplasms of the head and neck area or anus, among others [13,14]. In contrast, despite reaching high doses of radiation, poor clinical results are obtained for high-grade gliomas and the majority of locally advanced NSCLC. This poor therapeutic response is in part due to the extremely radio-resistant profile of these CSCs, and this explanation may be combined with the fact that larger lesions have higher failure rates, as in the case of lung cancer [15,16,17,18,19,20]. It has been suggested that larger tumors have more CSCs, allowing the appearance of a greater number of resistant clones and therefore a greater possibility of therapeutic failure [21,22].

In an attempt to show that higher total dose is associated with better disease control, the phase III clinical trial RTOG 0617 proposed two treatment arms for patients affected by locally advanced NSCLC, in which the 74 Gy arm (versus 60 Gy) obtained worse clinical results. This surprised the oncology community and was the result of a detrimental effect on the surrounding healthy tissues, which meant a deleterious effect on survival in patients treated with higher doses [23]. These factors that can determine different responses to radiation are general phenomena that have long been considered predictive of treatment response. Today, they are still far from being customizable elements and differ substantially between different histological subtypes, anatomical locations, and even between two tumors with similar characteristics. Although these factors are taken into account when considering the therapeutic approach with ionizing radiation, they are not sufficient, either individually or jointly, to predict therapeutic responses, nor do they fully explain the lack of efficacy of treatment in some patients.

### 1.3. Epidemiology and Classification of Lung Cancer

**Figure 1:**
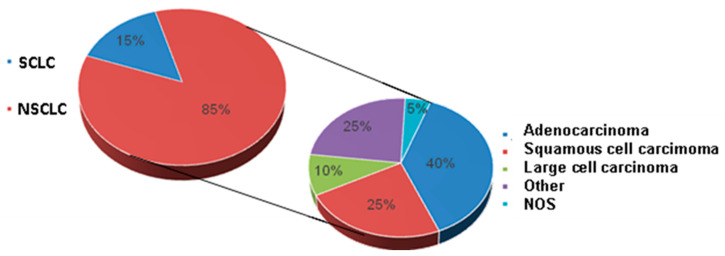
General classification of lung cancer and classification of the non-small-cell subgroup.

Lung cancer, due to its frequency and its impact on the lives of patients and on health systems, is one of the most important health problems in our society. It is the leading cause of cancer mortality worldwide. The incidence in the European Union is 288,100 new cases per year, with a mortality of 252,500 (181,900 men and 70,600 women) [24]. The main risk factor for its development is tobacco consumption [25,26,27,28]. Based on its histological characteristics, it is divided into two large groups: small cell lung cancer (small cell) and non-small-cell lung cancer (NSCLC). The latter accounts for approximately 85–90% of the total cases. In turn, this group is subdivided based on histological and molecular characteristics into adenocarcinomas (the majority), squamous, large cell, neuroendocrine, and NOS (Figure 1).

### 1.4. Therapeutic Approach to NSCLC

Currently, three clinical scenarios are considered for cancer treatment in newly diagnosed patients: resectable, locally advanced (unresectable) or metastatic. As shown in the main international therapeutic guidelines [29], radiotherapy treatment plays an important role in all scenarios. It is an alternative to surgical treatment in early stages (Stage I and II without nodal load) by stereotactic body radiotherapy (SBRT) and complementary after surgical resections with affected edges and/or positive nodal load (N2). In locally advanced stages (Stage III), where an initial surgical resection is not possible, normofractionated radiotherapy treatment together with chemotherapy treatment (concomitant or sequential) is the therapeutic standard. In patients with 1 to 5 thoracic and/or extrathoracic lesions (oligometastatic disease), the option of local treatment with radical intention is considered an effective alternative. Patients with a significant burden of systemic disease benefit from palliative radiotherapy treatment with analgesic, hemostatic or decompressive intent. Finally, the majority of patients with metastatic spread at the level of the central nervous system receive radiotherapy treatment in a protocolized manner, if their health status allows it. Focusing on the locally advanced unresectable scenario (Stages IIIA, IIIB and IIIC), there is currently no standard radiochemotherapy regimen, although the combination of a platinum-based scheme and thoracic radiotherapy has significantly improved the survival of these patients. These patients are treated with standard radiation doses of 60–66 Gy concurrently, or sequentially, with combined chemotherapy [30,31,32]. Fractionations may vary, but generally 1.8 to 2 Gy/fraction/day (normofractionated) is used. Despite the application of a combined cytotoxic treatment, we continue to observe local relapse rates of 30–50% in this group of patients [33,34]. This fact alone justifies the need to continue delving into the biological keys that govern the poor clinical results obtained to date.

### 1.5. Molecular Characterization of NSCLC and Its Impact on the Therapeutic Approach

Research focused on improving antineoplastic therapies in lung cancer has been based on the genomic and proteomic study of tumors with a known specific genetic basis, such as EGFR and KRAS mutations. This molecular classification has influenced the response to biological therapies based on monoclonal antibodies and tyrosine kinase inhibitors in patients with lung cancer [35,36,37]. On the other hand, a nonuniform response of patients to these therapies has been observed, proposing a resistance model that could be mediated by other mutations in some relevant genes (insertions in EGFR [38], KRAS [39], amplification of MET [40,41,42] or mutations in the kinase domain of HER-242). We also know that the tumor genetic profile has a relevant impact on the response to chemotherapy [43] and radiotherapy [44,45]. Multiple studies have related some of these mutations with mechanisms of radioresistance or radiosensitivity in NSCLC [45,46]. However, little is known about the role of these different radiotherapy fractionation schemes and their combination with other systemic or local treatments in the tumor response depending on the molecular profiles described [47,48].

## 2. Radiobiological Biomarkers

Several genes have been proposed as radiobiological markers. These include genes related to intrinsic radiosensitivity, such as EGFR, HER2, ALK; DNA repair such as ATM, XRCC1, RAD21, RAD50, and BRCA; genes related to repopulation and cell cycle redistribution, such as CD44, Ki67, and CDKN1A; and genes related to reoxygenation, such as HIF1α, HIF2α, VEGF [49,50,51,52]. However, no biomarkers are currently considered as conditioning factors for radiotherapy treatment, despite expanding knowledge that some mutations can affect the response to ionizing radiation. Thus, the classification of NSCLC based exclusively on clinicopathological characteristics has been the only determinant of the therapy administered.

**Figure 2:**
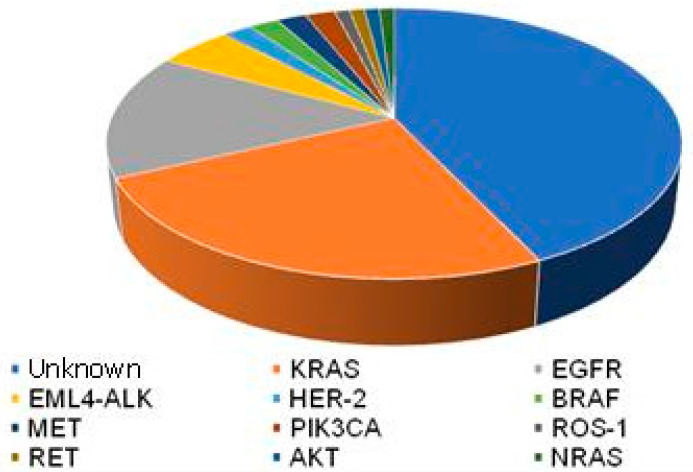
Relevant mutations in adenocarcinoma and their incidence. Extracted from [55,56].

In recent years, there has been a radical change in the way these tumors are classified [53,54]. The availability of biological material and advances in transcriptomic analysis techniques have made it possible to improve the subclassification of neoplasms encompassed within NSCLC. Likewise, advances in molecular biology and genetics have shown that some specific molecules contribute to the sporadic appearance of lung cancer and are useful as therapeutic targets and/or as predictive biomarkers of response [55]. In 2011, the results of the mutational study of genes responsible for lung cancer (EGFR, KRAS, BRAF, HER2, AKT1, PIK3CA, MEK1, EML4-ALK, MET) were published, identifying the existence of at least one mutation in the above genes in more than 60% of the samples studied (Figure 2). In more than 90% of cases, these mutations were considered to be exclusive, understanding this as the existence of a single mutation in a tumor sample [56].

The identification of these mutations led to the creation of molecular therapies aimed at improving survival in subgroups of patients with metastatic disease. Likewise, the role of these mutations in the response to ionizing radiation has been studied in depth, with the goal of offering combined treatments that can potentially improve the therapeutic results in NSCLC patients in early and locally advanced stages. This manuscript does not aim to delve further into the molecular alterations addressed by immunotherapy, or the potential synergies derived from its combination with radiotherapy treatment.

### 2.1. Role of the Epidermal Growth Factor Receptor (EGFR)

EGFR is an important regulator of the tumorigenic process, mediating the processes of proliferation, apoptosis, angiogenesis, and tumor invasion. It is amplified and/or overexpressed in up to 6% and mutated in 10–15% of NSCLC. Likewise, together with its ligands, it is constitutively activated during the initiation and progression of neoplasia. NSCLC cell lines harboring mutations in the EGFR tyrosine kinase domain show increased radiosensitivity compared to native EGFR cell lines. The radiosensitivity of both NSCLC cell lines with mutant EGFR and human bronchial epithelial cells that stably express mutant forms of EGFR has been attributed to various aspects: (1) delayed DNA repair kinetics, (2) defects in the STOPs induced by radiation during DNA synthesis or in mitosis and (3) an increase in both the apoptotic phenomenon and the appearance of micronuclei. Apparently, mutated EGFR is incapable of translocating to the nucleus, which makes it difficult for it to interact with DNA-dependent protein kinase (DNA-PK), a fundamental enzyme in the radiation-induced double-strand break repair process. Inhibition of EGFR by tyrosine kinase inhibitors (TKIs) or by monoclonal antibodies (mAbs) has shown limitations regarding radiosensitizing NSCLC cell lines in vitro and in vivo [57,58,59,60,61,62,63,64,65,66,67,68,69] 

### 2.2. Ras Family of Oncogenes: Role of KRAS in NSCLC and Their Response to Ionizing Radiation

The RAS family of oncogenes (HRAS, KRAS and NRAS) encodes signal transduction proteins that are related to the transmission of signals from extracellular growth receptors, such as EGFR. They are small GTP-binding proteins located on the inner face of the plasma membrane that have GTPase activity. After activation of RAS through the exchange of GDP for GTP, multiple downstream signaling effectors are activated, such as MAPK, STAT and PI3K, which ultimately regulate the phenomena of proliferation, motility, and apoptosis [70,71,72]. RAS activating mutations prevent the hydrolysis of GTP to GDP, leading to constitutive activation of the aforementioned signaling cascades. Approximately 30% of adenocarcinomas and 5% of squamous cell carcinomas have KRAS mutations. Although it is still controversial at present, the activation of KRAS mutations is a marker of poor prognosis in NSCLC. In 2011, Sun et al. published a study in which they evaluated the role of these mutations in tumor radioresistance [73]. The results showed that the lung cancer cell line HCC2429, in which KRAS mutated at position 12V had been transfected, showing a decrease in the apoptotic response after radiation. The same team also showed that the specific inhibition of JAK2 by the TG101209 molecule induces a radiosensitizing effect by inhibiting STAT3 phosphorylation and the consequent reduction in survivin expression in the HCC2429 and H460 cell lines [73]. Furthermore, in vivo experiments showed that survivin inhibition was associated with an increase in apoptosis, a reduction in tumor proliferation and associated vascular density. Once the protective effect of survivin is overcome, the differences observed in apoptosis between the two cell lines used in this study (H460 and HCC2429) seem to be explained by the mutational status of KRAS. Wang et al. proposed osteopontin (SPP1) and its relationship with EGFR-dependent mitotic-type chromatin condensation (MLCC), with a higher radioresistance profile in some NSCLC cell subpopulations with mutated KRAS [74]. Chromatin condensation has been related to increased protection against DNA double helix breaks and potentially with negative regulation of inhibitors of stem-like properties, such as invasion and metastasis [74]. In this way, a model is proposed in which the stem phenotype is connected to the EGFR and SPP1 pathways, cooperating to modulate chromatin condensation and the induction of double helix breaks. Mutated KRAS NSCLC cells with activation of the MLCC pathway and low levels of BIM are more prone to genomic alterations in the tumor suppressors TP53 and CDKN2A. Likewise, there seems to be a positive relationship between SPP1 levels and TP53 mutations. The identification of comutations in KRAS and TP53 has been related to higher levels of radioresistance in in vitro and in vivo models based on cell lines and xenografts of NSCLC, proposing the escalation of radiotherapeutic dose and/or radiosensitization by tyrosine kinase inhibitors as routes to overcome this resistance [75]

### 2.3. EML4-ALK

Fusion of the echinoderm microtubule-associated protein 4 (EML4) gene with the anaplastic lymphoma kinase (ALK) gene was first identified in neoplastic non-small-cell lung cancer cells [76]. The fusion is associated with the generation of a transcript translated into a protein (EML4-ALK) with a tyrosine-kinase domain, which promotes and maintains the malignant characteristics of the neoplastic cell. This fusion, the result of an inversion in the short arm of chromosome 2, occurs in approximately 5% of NSCLC, generally young patients, nonsmokers, and adenocarcinoma subtypes. Its existence is mutually exclusive with mutations in RAS and EGFR. Its function as an oncogene is dependent on its tyrosine kinase activity, which has made it possible to use treatments directed against this target (for example, crizotinib and ceritinib, among others). The clinical results derived from phase III trials have shown the superiority of crizotinib compared with chemotherapy in metastatic patients with ALK rearrangement, both in first-line and second-line therapy [77,78]. The majority of patients treated with crizotinib acquire resistance to the drug within the first 12 months of treatment [79]. In this sense, ceritinib, a second-generation ALK tyrosine kinase inhibitor, has shown an activity up to 20 times greater than crizotinib, demonstrating clinical responses in patients previously treated with crizotinib [80], as well as better results than standard chemotherapy in first- and second-line treatment [81,82]. On the other hand, these targeted treatments have shown activity against the tyrosine kinase function of MET and ROS1, whose amplification and rearrangement fusion, respectively, have been described in NSCLC. MET and ROS1 have been described as responsible for resistance in tumors with EGFR mutations that have acquired resistance to tyrosine kinase inhibitors (erlotinib, gefitinib) [83]. In 2013, Sun et al. demonstrated the radiosensitizing properties of crizotinib in an in vivo and in vitro model in ALK+ lines [84]. Analyzing the downstream ALK signaling pathway, they demonstrated activation of AKT, ERK, and STAT3 after irradiation and how combining crizotinib and radiotherapy completely inhibited ALK and decreased effector activation. Similar results have subsequently been obtained both with the combination of crizotinib and with the second-generation molecules of the same family, confirming its radiosensitizing effect to both photons and carbon ions. To date, some working groups have published their experience in patients treated with crizotinib and ablative radiotherapy techniques in patients with oligometastatic disease who progressed to targeted treatment. These studies proposed irradiation as a weapon to overcome resistance phenomena that appear, suggesting a benefit in terms of survival for patients who continued with targeted treatment [85,86]. Despite the accumulated scientific evidence on the impact of relevant mutations in NSCLC, none of them conditions radiotherapy treatment, obtaining clinical responses in tumors where systemic therapy has not been effective. This confirms the multitarget effect of radiation and its condition as a fundamental therapeutic weapon in all stages of the disease.

## 3. Gene Signatures with Prognostic and Predictive Response Value in NSCLC

Given the published scientific evidence, we believe that the search for prognostic and/or predictive gene signatures of response to radiotherapy treatment can significantly help clinical decision-making. These signatures may condition the fractions, the total dose to be administered and/or the combination of systemic treatments in conjunction with radiation. The ultimate goal is to achieve better clinical results, minimizing the adverse effects associated with current cancer therapies.

A gene expression signature can be defined as the specific pattern of expression of one or several genes with validated specificity in terms of diagnosis, prognosis, or prediction of response to some treatment. For more than 20 years, with the development and improvement of microarray technology, numerous studies have been generated, based mainly on transcriptomic analysis, proposing different gene signatures as prognostic biomarkers in adenocarcinoma [87,88,89,90,91,92,93,94,95], squamous cell carcinoma [96,97,98] and NSCLC in general [99,100,101,102,103,104,105,106,107,108,109,110]. Some of these studies have tried to identify prognostic and predictive biomarkers of response to systemic treatments. Most of them focused on the identification of markers that could help in clinical decision-making about the suitability of administering adjuvant systemic treatment in early stages of NSCLC after surgery [111,112]. This is based on the management of patients affected by breast cancer, in which there are already several gene platforms that help predict survival and the potential benefit of administering adjuvant chemotherapy [113]. In contrast, very few published studies have suggested predictive signatures of response to ionizing radiation in NSCLC.

### 3.1. NSCLC Gene Signatures

In 2002, Beer and collaborators [87] published one of the pioneering articles on the generation of gene signatures with prognostic capacity. They proposed a gene profile of 50 genes, which allowed the identification of a subgroup of patients within stage I whose behavior and survival are similar to those included as stage III. They proposed that modification of the therapeutic approach in these patients could lead to an improvement in their prognosis. They exclusively took samples of adenocarcinomas, mainly in early stages. Most of the patients did not receive adjuvant treatment. In 2004, Tomida et al. [114] proposed a prognostic signature of 25 genes resulting from the analysis of a total of 8644 genes in tumor samples from 50 patients who underwent surgery for NSCLC: 30, 16, and 4 samples corresponded to the adenocarcinoma, squamous cell carcinoma and large cell carcinoma subtypes, respectively. Regarding staging, 23, 11, and 16 patients were classified as stage I, stage II, and stage III, respectively. They carried out several subanalyses by histology, proposing a 16-gene signature for a better prognostic classification in the squamous subtype and another 12-gene signature for the adenocarcinoma subtype. These genes came from the proposed common signature of 25, without distinction between histological subtypes. They concluded that their study was partially robust (mainly limited by a small sample size) and that these signatures were independent of the tumor, nodes and metastasis staging system (TNM) at diagnosis. In 2006, Raponi et al. published a study focused on the identification of prognostic subgroups in the squamous subtype of NSCLC [96]. They used 129 samples of squamous cell carcinoma (the majority from patients with stage I disease) and carried out a microarray analysis with validation by RT–PCR and immunohistochemistry. They obtained a group of 50 genes with the capacity to separate the 129 samples of squamous cell carcinoma by prognostic subgroups (prognostic classifier). Subsequently, validation was carried out in another independent cohort with 36 samples (mostly from patients in stage I). Likewise, jointly using this set of genes, those obtained in a study focused on the adenocarcinoma subtype and published by the same authors tested the set of 100 genes in a cohort with 52 samples (50% squamous and 50% adenocarcinoma), with the aim of establishing a valid prognostic signature for the two main histological subtypes in NSCLC. They concluded that the classifier gene set maintained its ability to separate prognostic subgroups in the adenocarcinoma sample but not in the squamous subtype. Thus, new validations are necessary in larger and more homogeneous cohorts.

In 2007, Lau et al. [115] published a prognostic signature of three genes focused on NSCLC in early stages. The objective of this study was to validate a signature with the capacity to identify, within the initial stages of the disease, those patients with differential prognostic profiles. The genes proposed in the signature were STX1A, CCR7 and HIF1A. In 2007, Chen et al. [116] published a prognostic signature composed of five genes (DUSP6, MMD, STAT1, ERBB3 and LCK), which were the result of a combined analysis of microarrays and RT–PCR in tumor samples from 101 surgically treated patients (mixed adenocarcinoma subtype, squamous and others). Subsequently, these results were validated in an independent cohort of 60 patients and in a microarray set of 86 patients. In the multivariate analysis considering other clinical variables, such as age or stage, the proposed signature maintained its statistical significance. On the other hand, this gene signature predicted RFS in a statistically significant way in the initial cohort of the study, a result that was not shown in the validation cohorts [116]. More recently, Zuo et al. [117] proposed a signature of six genes with the capacity to predict disease-free survival and overall survival in NSCLC, without discriminating by histological subtypes. They were based on the combination of genetic information from three public databases that encompass all histological subtypes (mainly adenocarcinomas). After obtaining the candidate genes with prognostic capacity, validation was carried out with the TCGA lung cancer cohort. The proposed signature was comprised of the PLEKHH2, ISCU, CLUL1, CHRDL1, PAIP2B, and CDCP1 genes. In the same year, the same team published another article in which they proposed an 8-gene prognostic signature for patients with early-stage NSCLC [118]. The proposed genes (CDCP1, HMMR, TPX2, CIRBP, HLF, KBTBD7, SEC24B-AS1, and SH2B1) do not coincide with those proposed in other studies. This may also be due to multiple causes: (1) the signature was based on the identification of genes with prognostic value (HR< o >1 and *p* < 0.05) common in four different public databases, and (2) they exclusively selected he samples that corresponded to the early stage of disease.

### 3.2. Ionizing Radiation-Based Gene Signatures

Unlike the works presented above, articles published on the identification of predictive or prognostic gene markers which focused on cohorts whose main treatment was ionizing radiation in the case of NSCLC are scarce. Based on research carried out by the American National Cancer Institute (NCI) on in vitro analysis of the biological effects of different antineoplastic drugs on cell lines [119], Torres-Roca et al. published in 2005 [120] the identification of genes correlated with the response to ionizing radiation in 35 cell lines of the NCI-60 panel, representative of nine types of cancer. They evaluated genetic contributions to radiosensitivity by quantifying the surviving fraction after exposing cell lines to a standard radiation dose of 2 Gy (the clonogenicity assay). They concluded that their response predictor model based on gene expression could be very useful to improve the therapeutic approach of patients, assuming that the model would require in vivo validation [120]. Subsequently, Eschrich et al. extended the model to 48 cell lines from the NCI panel and included other biological variables, such as the mutational status of KRAS and TP53, as well as the tissue of origin [121,122]. Combining these data, they created a linear rank-based algorithm to calculate a radiosensitivity index (RSI). The RSI has since been validated in multiple cohorts of patients with different neoplastic entities (pancreas, glioblastoma, liver, brain and lung metastases, breast cancer) [123,124,125,126,127,128]. The RSI forms a predictive signature of response to radiotherapy treatment composed of the genes AR, cJUN, STAT1, PKC, RELA, ABCc, SUMO1, CDK1, HDAC1 and IRF1. 

Almost simultaneously with these works, in 2008, Amundson et al. [129] published an article in which they analyzed the genetic response to stress produced by ionizing radiation in a panel of 60 NCI cell lines and in three of their own cell lines. This work, contrary to those carried out previously, analyzed the changes in gene expression induced by radiation, not as a function of the basal gene profile. Unlike what was found when analyzing the basal expression levels, no significant differences were observed in the levels of gene expression depending on the tissue of origin (ovary, lung, breast), except in the case of the cell lines derived from lymphoid/myeloid tissue. This could support the hypothesis of the existence of molecular determinants of sensitivity/resistance to radiation that would be common to all tumor subtypes (not including hematological neoplasms). They concluded that, although there are changes in gene expression induced by radiation, especially in the p53 pathway, basal gene expression levels may be better predictors of response. 

One of the most important studies in the field of radio-oncology and physical and molecular radiobiology was published in 2017 by Scott et al. [130]. This article proposed a model to adapt the radiotherapy prescription to the individual sensitivity of each patient’s tumor. The model, called GARD (genome-based model for adjusting radiotherapy dose, of its acronym in English), combines the information derived from the radiosensitivity index (RSI) and the linear quadratic model (LQ model), which proposes the existence of two parameters that impact the cytotoxic capacity of radiation, one of them being proportional to the dose of radiation administered (factor α) and the other being proportional to the square of the dose (β-factor). This mathematical model has been used for decades to calculate the equivalent biological dose of different radiotherapy treatment schemes, considering the α/β ratio of each tumor, which has been used to propose altered radiotherapy fractionations. This has made it possible to achieve biologically equal or superior results to treatments based on daily normal fractionation (1.8/2 Gy/fraction) [131]. This extensive work uses multiple cohorts of different neoplastic histologies (breast cancer, esophagus, head and neck, stomach, cervix, gliomas, pancreas, lung, nonmelanoma skin cancer and melanoma) and establishes a numerical value for GARD (normally in the range of 1 to 200), relating a higher level of GARD with a greater therapeutic effect of radiotherapy treatment. More recently, the same group has proposed GARD as a pan-cancer predictor of radiation benefit (with impact in OS and RFS), based on a cohort-based pooled analysis of more than 1500 patient representatives of several cancer subtypes, including NSCLC [132]. As an example of the current interest in this field, Ma and collaborators [133], using LASSO Cox regression analysis in the TCGA NSCLC database, demonstrated that eight genes (BLACAT1, ALPP, SLC6A11, IGFN1, HIST1H2BH, KCNJ12, FOLR3, and RPS4XP22) based on risk score could predict the prognosis of NSCLC patients with or without radiotherapy treatment. However, in this work, 365 genes potentially correlated with the radiotherapy response were also described, and the original dataset encompassed almost 1000 patients where only 127 received radiotherapy. Focused on the TCGA lung cancer database, our group have recently published a prognostic of a predictive radiation-based 6-gene signature derived from the differentially expressed genes according to the radiophenotype of NSCLC cell lines and applied to a 107-patient cohort of stage I-III NSCLC, treated with radiation and other therapies [134]. A summary of the gene signatures analyzed in this review is shown in Table 1. 

Besides gene expression signatures, it is important to comment on other studies that consider several factors which have classically been used to predict response to treatment and vital prognosis, both in lung cancer and in other solid and hematological neoplasms. Some of these factors are the general condition of the patient, tumor size, nodal load, age, the existence of comorbidities, previous treatments administered and TNM classification, along with other elements identified in blood samples (inflammatory markers, such as interleukins and C-reactive protein; indirect markers of hypoxia, such as osteopontin, carbonic anhydrase IX and lactate dehydrogenase; or indirect markers of tumor burden, such as carcinoembryonic antigen or cytokeratin 21-1 fragments) [135,136,137,138,139,140,141,142,143,144]. In this way, Dehing-Oberije et al. published the results of their study, in which they developed and validated a prognostic signature in patients with NSCLC treated with radiotherapy +/− chemotherapy. It combined the determination of biomarkers in peripheral blood such as carcinoembryonic antigen (CEA) and interleukin 6 (IL-6)) with other clinical factors, such as sex, general condition of the patient, forced expiratory volume (FEV1), number of affected lymph nodes and primary tumor volume (GTV) [145].

### 3.3. microRNAs

There are high potential predictive and prognostic roles of certain microRNAs (tumor and circulating). There is a wide field of research focused on the function of this noncoding RNA that potentially regulates gene expression. Some of the more than 2000 miRNAs identified to date can be considered diagnostic and prognostic biomarkers in many neoplastic entities, including NSCLC [146,147,148,149]. In 2010, Hu et al. [147] identified a prognostic signature in NSCLC composed of four miRNAs. They included study samples from patients in early and locally advanced stages (stage I to III) who had been treated with surgery and chemotherapy. Apparently, no patient received radiotherapy treatment. One of the most interesting studies is that published by Sun et al. in 2018, in which they proposed the role of certain circulating miRNAs (c-miRNAs) in association with other clinical factors as determinants of response to radical doses of ionizing radiation in NSCLC. To do this, they proposed the generation of a scale or a score based on the radiation dose and the objective response, which they called “DRS” (Dose Response Score, of its acronym in English). The cohort included patients in several clinical trials in which radiation dose escalation was considered in stages II and III (more than 90% were stage III). They proposed a total of 11 c-miRNAs, which, together with variables such as stage, age, radiation dose administered, systemic treatment and the Karnofsky general status scale, were used to determine the DRS of each patient. The results showed that those patients who had a low DRS benefited from high doses of radiation, with an impact on survival, represented by Kaplan–Meier curves. In contrast, patients with a high DRS did not show differences in survival depending on the dose administered. Likewise, they demonstrated that a low DRS value in patients treated with high doses made it possible to predict a lower risk of metastasis. In contrast, they could not demonstrate a statistically significant predictive capacity for local control of the disease [150].

## 4. Limitations

As reflected in most of the works published to date focused on biomarkers, taken individually or in the form of gene signatures with potential prognostic and/or predictive capacity, the main problem is the difficulty in extrapolating in vitro data to the clinic. The heterogeneity of samples, the different extraction techniques of genetic material and the constant development in the biostatistical and bioinformatic approaches make it very difficult to compare pioneering with more recent studies. These works with potential translational capacity generally showed several limitations: 1—The sample size of the discovery set limited the statistical power of the bioinformatic analysis. 2—The evaluation of the response to ionizing radiation as the standard for the determination of response. As reflected in the literature [121,151,152,153], there are many differences in the published values of dose, treatment types and efficacy, which implies potential bias when establishing classifications in radiosensitive and radioresistant, and this can condition the supervised bioinformatic analysis. 3—In the case of locally advanced NSCLC, on many occasions there is not enough biopsy material to expand the battery of mutational determinations or to perform tissue microarrays or immunohistochemical validation techniques. 4—Most of the public databases with bioinformatic information on NSCLC feed on patient samples mainly in localized and/or metastatic stages, which generally have not received or do not reflect information on radiotherapy treatment; this has greatly limited the sample size used in the studies, as well as the ability to obtain additional cohorts for further validation. 5—The generation of prognostic and/or predictive gene signatures of response to some treatments do not usually assess other biological factors not directly related to the biology of the tumor itself. These factors, whose genetic and epigenetic bases can condition the response to certain oncological treatments and even significantly condition the overall survival of patients, are undoubtedly the greatest biases when giving translational value to these signatures.

The generation of new gene signatures with prognostic and/or predictive capacity in pathologies such as NSCLC can greatly benefit patients. Knowledge about the patient’s responder profile or vital prognosis prior to starting treatment can help us optimize the therapeutic approach and avoid dreaded and frequent iatrogenesis. However, most of the studies may need additional validation steps before entering the clinic, measuring the relative contribution of each of the proposed genes to the predictive value and organizing a prioritization algorithm for the genes of any given signature.

## 5. Conclusions

NSCLC is the leading cause of cancer-related death in men and the second in women worldwide [24]. A high percentage of patients are diagnosed in locally advanced and unresectable stages, and the majority (including those diagnosed in early operable stages) succumb to metastatic dissemination. In such a situation, the 5-year prognosis remains bleak. Currently, the standard treatment in the initial stages is surgery with or without systemic treatment and/or radiotherapy. In more advanced, nonresectable but nonmetastatic stages, the standard treatment continues to be combined radiochemotherapy regimens (platinum-based). In metastatic stages at diagnosis, treatment consists of chemotherapy or treatments directed at specific molecular targets, with or without the addition of radiotherapy treatment in some cases, all for palliative purposes. Currently, the tumor staging system by TNM classification continues to be the most powerful instrument for predicting patient survival and is the axis on which the oncology community proposes to focus the therapeutic approach for NSCLC and most neoplasms [29,154,155]. Despite efforts to obtain clinical, pathological and/or molecular information that could be used to predict response to treatment and improve prognostic capacity, there are currently no validated biomarkers in NSCLC that enhance decision-making regarding individualized treatment selection in the nonmetastatic setting. There are multiple proposals for gene signatures that attempt to predict survival or predict response to treatment (not radiotherapy), but they mainly focus on early stages or metastasis at diagnosis. In contrast, there have been few studies that raise these predictive and/or prognostic elements in nonmetastatic locally advanced stages, where treatment with ionizing radiation plays an important role. The radiation oncologist, in particular, lacks molecular markers that serve to condition the radiotherapy treatment beyond the general recommendations, for example, from the pathology report, considering the situation of surgical margins, or the positive nodal load [29,154]. One of the difficulties faced in the identification of predictive and prognostic signatures in NSCLC is the inability to identify whether the clinical, therapeutic, histological, or molecular variables have the same weight when conditioning the sustained therapeutic response and overall survival. This is reflected by Subramanian and Simon, in the 2010 publication, where biases presented by the different gene signatures proposed led to the conclusion that these biases and the poor design of prognostic and predictive studies limit the inclusion of the results into daily clinical practice [156].

## Figures and Tables

**Table 1:** A summary of the gene signatures analyzed in this review.

Author/Year	Gen Expresion Signature	Cancer Type	Histologies	Number of Patients /TNM Stage	Prognostic Value?	Predictive of Response Value?	Used in the Clinic?	FoYes, It Is a NOcused on Radiotherapy	Reference
Beer et al./2002	50 genes	NSCLC	ADC	67/I; 19/III	Yes	No	No	No	[87]
Tomida et al./2004	25 genes (all subtypes)/12 genes (SCC)	NSCLC	ADC/SCC/LCC	50/I	yes	No	No	No	[114]
Lau et al./2007	STX1A, HIF1A, CCR7	NSCLC	ADC/SCC	92/I; 36/II; 17/III	yes	no	No	No	[115]
Chen et al./2007	DUSP6, MMD, STAT1, ERBB3, LCK	NSCLC	ADC/SCC/other	59/I-II; 42/III	yes	yes	No	No	[116]
Zuo et al./2019	PLEKHH2, ISCU, CLUL1, CHRDL1, PAIP2B, CDCP1	NSCLC	ADC/SCC	410/I; 220/II; 109/III; 22/IV	yes	yes	No	No	[117]
He et al./2019	CDCP1, HMMR, TPX2, CIRBP, HLF, KBTBD7, SEC24B-AS1, SH2B1	NSCLC	ADC/SCC/LCC/other	923/I; 417/II	yes	no	No	No	[118]
Scott et al./2017–2021	AR, cJUN, STAT1, PKC, RELA, ABCc, SUMO1, CDK1, HDAC1, IRF1 (GARD and RSI)	NSCLC and others	NR	60/III	yes	yes	No	Yes	[130,132]
Ma et al./2019	BLACAT1, ALPP, SLC6A11, IGFN1, HIST1H2BH, KCNJ12, FOLR3, RPS4XP22	NSCLC	ADC/SCC	509/I; 277/II; 163/III; 32/IV; 12/NR	yes	yes	No	Yes	[133]
Peinado-Serrano et al./2022	APOBEC3B, GOLM1, FAM117A, KCNQ1OT1, PCDHB2, USP43	NSCLC	ADC/SCC	57/I-II; 50/III	yes	yes	No	Yes	[134]

TNM: tumor, nodes, metastasis; NSCLC: non-small cell lung cancer; ADC: adenocarcinoma; SCC: squamous cell carcinoma; LCC: large cell carcinoma; GARD: genome-adjusted radiation dose; RSI: radiation sensitivity index.
